# Evaluation of Risk Factors of Peri-Implant Disease Using a New Manual Risk Assessment Model: A Clinical study

**DOI:** 10.1155/2022/1347569

**Published:** 2022-10-07

**Authors:** Mahima Jain, Mohd Shafiq Bin Shaffa'ee, Ashita Uppoor, Swati Pralhad, Sangeeta U. Nayak, Sharon Saldanha

**Affiliations:** ^1^Manipal College of Dental Sciences, Mangalore, Manipal Academy of Higher Education, Manipal-575001, India; ^2^Department of Periodontology, Manipal College of Dental Sciences, Mangalore 575001, Manipal Academy of Higher Education, India; ^3^Department of Prosthodontics and Crown and Bridge, Manipal College of Dental Sciences, Mangalore, Manipal Academy of Higher Education, Manipal 575001, India

## Abstract

**Background:**

Implants are being widely used as a treatment option and are considered the best line of treatment owing to their high level of predictability. However, over 5 years, 0–14.4% of dental implants have demonstrated peri-inflammatory reactions associated with loss of crestal bone and ultimately loss of the implant. Peri-implant diseases are categorized into peri-implant mucositis and peri-implantitis. There are a number of risk factors associated with these conditions, and the early detection of these possible risk factors, change in the treatment protocol whenever required along with a regular follow-up, can ensure a better survival rate of dental implants. In the present study, an attempt has been made to evaluate the risk factors associated with peri-implant diseases and also to correlate these risk factors to the prevalence of peri-implant diseases using the formulated risk assessment model. *Methodology*. The risk assessment model was prepared based on existing literature explaining the risk factors for peri-implant diseases. This study was conducted as a pilot study, and the method of complete sampling was used wherein all subjects in whom implants have been placed at the Department of Periodontology and Department of Prosthodontics were recalled and assessed. The model was then evaluated on patients in whom dental implants were placed and the implants were loaded for a year. A total of 13 subjects with 21 implants were assessed for the presence or absence of risk factors, and a score was given. Test of proportion and chi-square test was done. *Results and Discussion*. Of the 21 implants assessed, 15 implants were found to be at low risk and 6 implants with moderate risk of peri-implant disease. The number of implants with low risk was higher in implants with peri-implant mucositis (25%) whereas the implants with moderate risk was higher in implants with peri-implantitis (75%). This comparison was statistically significant with a *p* value of 0.022.

**Conclusion:**

This risk assessment tool can be used in the early detection of peri-implant disease, and identifying the risk factor may help in the success rate of the implant survival.

## 1. Introduction

The use of dental implants has changed the treatment of partially and fully edentulous patients today. To manage a broad range of clinical dilemmas, implants have become the best line of treatment approach. This is due to their high level of predictability and their ability to be used for a wide variety of treatment options [1].

Over 5 years, 0–14.4% of dental implants have demonstrated peri-inflammatory reactions associated with the loss of crestal bone and ultimately loss of implant [2].

Peri-implant diseases are categorized into peri-implant mucositis and peri-implantitis. Peri-implant mucositis is defined as the presence of inflammation confined to the soft tissues surrounding a dental implant with no signs of loss of supporting bone. On the other hand, peri-implantitis is characterized by an inflammatory process around the implant which includes both soft tissue inflammation and progressive loss of supporting bone beyond biological remodeling [3–6].

Etiological factors of peri-implant disease include bacterial flora which is similar to periodontitis (*Porphyromonas gingivalis*, *Prevotella intermedia*, *Aggregatibacter actinomycetemcomitans* etc.), biomechanical factors such as occlusal overload, systemic diseases such as diabetes mellitus, social factors such as poor oral hygiene and smoking, parafunctional habits such as bruxism, and iatrogenic factors such as lacking primary stability and premature loading during healing period [3].

Clinical features of peri-implant diseases include bleeding on probing, suppuration, probing depth more than 5 mm, and progressive bone loss beyond remodeling in cases of peri-implantitis. Normal expected bone loss is around 2 mm postimplant placement [4].

Numerous risk factors have been taken into consideration pertaining to peri-implant diseases, such as previous periodontal diseases and patient compliance, plaque control, uncontrolled diabetes mellitus, smoking, implant characteristics, occlusal overload, residual cement, presence of keratinized mucosa, and other potential risk emerging factors such as alcohol consumption and increasing the time of loading [7].

It has been suggested that these risk factors can be used to determine the emergence of peri-implant diseases that could consequently lead to implant failure.

Currently, the pervasiveness of peri-implantitis has increased exponentially as compared to the past decade: previously, in its budding stages as being developed as a viable treatment modality, dental implants could lead to failure and subsequent disease in a range of 1–47% [8], which subsequently increased to as high as 85% [9].

Early detection of these possible risk factors, change in the treatment protocol whenever required along with a regular follow-up, can ensure a better survival rate of dental implants. Various risk assessment tools have been applied and tried for patients in supportive periodontal therapy [10–12]. Of the assessment formats that have been proposed in this time , the periodontal risk assessment (PRA) [11] is the most extensively examined, applied, and researched into. Owing to the increased attention garnered by the growing incidence of peri-implantitis and peri-implant mucositis, and the focus on its pathobiology, it can be safely stated that scrutinizing and elucidating the risk probability of such conditions is the need of the hour, moving forward.

In 2020, Mayfield et al. [13] developed new a risk assessment tool “Implant Disease Risk Assessment (IDRA)” for peri-implant disease assessment.

In the present study, an attempt has been made to evaluate the risk factors associated with peri-implant diseases and also to correlate these risk factors to the prevalence of peri-implant diseases using the formulated risk assessment model.

## 2. Materials and Methods

The risk assessment model was prepared based on existing literature explaining the risk factors for peri-implant diseases. The parameters considered were suppuration, previous history of periodontal disease, plaque status using the modified plaque index, gingival status using the modified gingival index, diabetes state, smoking, residual cement, occlusal overload, width of the keratinized gingiva, and other factors like alcohol consumption.

Each of the abovementioned parameters was assessed as being present/absent or graded according to the existing index. Risk percentage was given to each of the options, and they ranged from 0–12.5%. The details are mentioned in Table 1.

The risk percentage for an individual was obtained by calculating the sum of the individual risk percentages for each parameter assessed. The individual was said to be at low risk when the risk percentage was less than 25%; moderate risk, between 25 and 50%; and high risk, when the percentage was more than 50%.

Once this model was prepared, the relationship between risk factors and prevalence of peri-implant disease was evaluated among the patients in whom dental implants were placed. To carry out this clinical evaluation, the institutional ethics committee approval (17100) was obtained. All the included patients were required to sign an informed consent form.

A method of complete sampling was used, wherein all subjects in whom implants have been placed at the Department of Periodontology and Implantology and Prosthodontics at MCODS, Mangalore, were recalled and assessed. This pilot study was timebound, and hence, only those implants which were loaded for more than a year, during the present study time, were included. Individuals who were partially edentulous and had an osseo-integrated dental implant placed (MIS system) and who were loaded for at least 1 year were taken into consideration. A total of 13 subjects with 21 implants were assessed.

All the clinical assessments were carried out by M. J. (Mahima Jain) and S. P. (Swati Pralhad). The examiners were trained in this field to avoid errors.

The demographic data were then recorded using questionnaires pertaining to the subject case history before clinically assessing them.

The subjects were then evaluated for the presence of peri-implant diseases by clinical and radiographic means.

The clinical parameters were assessed using a teflon-coated periodontal probe designed for use in implants, and the subjects were clinically assessed for plaque status using the Modified Plaque Index by Mombelli and Lang [3], gingival status using the Modified Gingival Index (MGI) by Mombelli and Lang [3], suppuration, and probing depth (assessed by probing peri-implant tissue at six aspects around the dental implant, namely, mesiobuccal, midbuccal, distobuccal, distolingual, midlingual, and mesiolingual aspects and the mean average probing depth was recorded), the width of keratinized gingiva and the presence of occlusal overload indicated by the presence of wear facets or premature contact were noted.

The radiographic assessment was carried out from the intraoral periapical radiograph (IOPA-R) taken using the paralleling technique—RINN XCP. The presence/absence of residual cement and crestal bone loss was assessed which was measured by taking linear measurement in a vertical direction using fixed reference points (CEJ of 2 adjacent teeth; draw a line correctly joining these 2 points; the distance between this line and deepest point of the crest of the residual ridge).

Based on the data recorded and the clinical findings, the subject was classified into three categories: healthy peri-implant tissue, peri-implant mucositis, and peri-implantitis (Table 1).

Data regarding the questionnaires and the clinical assessment were incorporated into the new risk assessment model (Table 2) for peri-implant disease, and the scores were tabulated. Based on the interpretation, the patients were categorized as low risk: <25%; moderate risk: >25%; and high risk: <50.

Chi square test and test of proportion were done to evaluate the various risk factors.

## 3. Results

The evaluation and correlation were based on the evaluation of 13 patients with 21 implants. Of the 13 patients, 5 were females and 8 were males. The average age of the patients was found to be 48.5 ± 1.254 years.

The history of the patients revealed that none of them were diabetic. However, 1 patient was found to be a current smoker, and 1 patient was a former smoker. Occasional alcohol consumption was mentioned by a few patients.

The plaque and gingival status of the implants were assessed. Of the 21 implants, among 42.9% (*n* = 9) implants, plaque could be recognized by running a probe. The abundance of the soft matter was seen among 9.5% (*n* = 2), and plaque could be seen by the naked eye among 28.6% (*n* = 6).

Isolated spots of bleeding were seen in 4 implants (19%), confluent bleeding was seen in 6 implants (28.6%), and 11 implants (52.4%) did not show bleeding on probing.

Suppuration, which is a sign of active disease when checked: no implants were seen to be positive for the same.

The width of the attached gingiva was recorded to be sufficient in 17 implants (76.2%), whereas it was not sufficient in 5 implants (23.8%).

Occlusal overload was present in only 2 implants (9.5%), and no implant was recorded with residual cement.

On the basis of all the abovementioned risk factors, each implant was categorized into healthy peri-implant tissue, peri-implant mucositis (*N* = 17), and peri-implantitis (*N* = 4).

In comparison between the implants with mucositis and implants, 41.2%, i.e., 7 implants placed in females were found to have peri-implant mucositis, and among the 14 implants being assessed in the male patients, 10 (58.8%) of them showed peri-implant mucositis, while the remaining 4 implants (100%) were recorded with peri-implantitis amongst which 2 implants had a history of smoking.

In 75% (*N* = 3) implants with peri-implantitis, plaque could be seen with the naked eye, whereas plaque could be recognized by running a probe in 47.1% (*N* = 8) of implants with peri-implant mucositis.

Of the implants with peri-implantitis, 2 implants (50%) showed the presence of isolated bleeding spots and 2 implants (50%) with confluent bleeding, whereas among the implants with peri-implant mucositis, 11 implants (64.7%) did not present with any bleeding on probing.

In comparison with the implants with peri-implantitis and peri-implant mucositis, taking into account all the abovementioned risk factors, there were 15 implants with low risk and 6 implants with moderate risk of peri-implant disease. The number of implants with low risk is higher in implants with peri-implant mucositis (25%), whereas the implants with moderate risk are higher in implants with peri-implantitis (75%). This comparison is statistically significant with a *p* value of 0.022 (Table 3).

## 4. Discussion

In the present study, the relationship between risk factors for peri-implant diseases and the prevalence of peri-implant disease was assessed using the new risk assessment model. This model was made based on literature regarding the risk factors for developing peri-implant diseases. The factors considered were past history of periodontitis/patient compliance, plaque status, gingival status, diabetes, smoking, residual cement, occlusal overload, the width of keratinized gingiva, and other risk factors like alcoholism [5, 6]. The data obtained for the patient was recorded in a clinical data sheet to help formulate diagnosis and risk assessment. Based on the presence or absence of risk factors and grades for indices, a classification of healthy peri-implant tissue, peri-implant mucositis, and peri-implantitis was made. The risk percentage was recorded for each of the parameters assessed, and the total risk percentage was calculated. A person was said to be at low risk, if the risk percentage was <25%, moderate risk if between 25 and 50%, and severe if >50%.

The need for a risk assessment model for peri-implantitis is based on the etiological and pathological similarities with periodontitis. An increase in the understanding of the etiology and pathogenesis of peri-implantitis triggered the need for an assessment model which could help in preventing peri-implantitis. The risk factors considered in the present study are based on the proven risk factors for periodontitis, with the inclusion of occlusal overload and residual cement [2].

Occlusal overload has been considered to be positively associated with peri-implant marginal bone loss [14]. This is due to the lack of periodontal ligament which makes nonaxial forces on the implant nonfavorable. Occlusal load in the present study has been evaluated clinically by the presence of occlusal wear facets and/or premature contacts on the implant prosthesis.

The presence or absence of residual cement was another factor considered. This was based on studies reporting incomplete removal of cement in the subgingival space because of the implant position and the superstructure design which hampers subgingival plaque removal and thus makes plaque removal difficult leading to inflammation [15, 16].

The Implant Disease Risk Assessment (IDRA) tool has been developed by Mayfield et al. [13]. The IDRA tool involves constructing a diagram using the eight risk factors for peri-implantitis. Based on the number of parameters in the low, moderate, and high-risk category, the patients are categorized as being low, moderate, or high risk IDRA patients. The rate of bone loss with age and prosthesis cleanability are two additional risk factors considered in IDRA. On the other hand, the risk assessment model developed by us evaluates each of the risk factors individually, and a risk percentage for the individual implant is obtained. Few additional factors considered by us are occlusal overload, the width of keratinized gingiva, and alcohol consumption.

However, the present model was used to determine the risk assessment in a smaller population, and hence, a similar study with a larger population can be carried out. With regard to the assessment, all the measurements and assessments were done manually and took approximately 15 minutes/patient. It is thus felt that a digital radiographic system can be used to assess the radiographic features, which would significantly reduce the time involved.

## 5. Conclusion

Based on the present study data, it can be concluded that this risk assessment tool can be used in the early detection of peri-implant disease, and identifying the risk factor may help in the success rate of the implant survival. The susceptibility of subjects before implant placement can be done using this risk assessment tool. More studies need to be carried out using this risk tool and compared it with other available risk assessment tools.

## Figures and Tables

**Table 1:** Clinical and radiographic parameters for healthy and diseased peri-implant tissue.

Healthy peri-implant tissue	Peri-implant mucositis	Peri-implantitis
Inclusion criteria		
(i) MGI score of 0	(i) MGI score of 1 or more	(i) MGI score of 1 or more
(ii) MPI score of 0	(ii) MPI score of 1	(ii) MPI score of 2 or more
(iii) Absent of suppuration	(iii) Present of suppuration	(iii) Present of suppuration
(iv) Probing depth of less than 5 mm	(iv) Probing depth of more than 5 mm	(iv) Probing depth of more than 5 mm
(v) No crestal bone loss	(v) Crestal bone loss less than 2 mm	(v) Crestal bone loss of more than 2 mm

MGI: modified gingival index; MPI: modified plaque index.

**Table 2:** New risk assessment model.

Previous periodontal history and compliance to therapy	Score	Previous periodontal history	Compliance	Risk percentage (%)
	0	Absent	Compliance/present	0
	1	Absent	Noncompliance/absent	2.5
	2	Present	Compliance/present	7.5
	3	Present	Noncompliance/absent	12.5

Plaque status	Score	Modified plaque index		
	0	No detection of plaque		0
	1	Plaque only recognized by running a probe across the smooth marginal surface of the implant		2.5
	2	Plaque can be seen by naked eye		5.0
	3	Abundance of soft matter		7.5

Gingival status	Score	Modified plaque index		
	0	No detection of plaque		0
	1	Plaque only recognized by running a probe across the smooth marginal surface of the implant		2.5
	2	Plaque can be seen by naked eye		5.0
	3	Abundance of soft matter		7.5

Diabetes status	Score	Blood sugar level (RPG)/mg/dl		
	0	<102		0
	1	102–109		2.5
	2	110–117		5.0
	3	118–125		7.5
	4	126–133		10.0
	5	>133		12.5

Smoking status	Score	Cigarettes/day		
	0	Nonsmoker		0
	1	Former smoker		2.5
	2	<10		5.0
	3	10–19		7.5
	4	20		10.0
	5	>20		12.5

Residual cement	Score	Residual cement		
	0	Absent		0
	1	Present		12.5

Occlusal overload	Score	Occlusal overload (either wear facet and/or premature contact)		
	0	Absent		0
	1	Present		12.5

Width of keratinized gingiva	Score	Width of keratinized mucosa		
	0	Sufficient (>2 mm)		0
	1	Insufficient (<2 mm)		12.5

Other potential risk factors	Score	Other risk factors (alcohol consumption)		
	0	Absent		0
	1	Present		10.0

**Table 3:** Chi square test with various risk factors involved in peri-implant disease.

		*N*	Diagnosis				Chi square	*P* value
			Implantitis		Mucositis			
			Count	%	Count	%		
Sex	F	7	0	0.00	7	41.20	2.471	0.116
	M	14	4	100.00	10	58.80		

Suppuration	Absent	21	4	100.00	17	100.00		
	Present	0	0	0.00	0	0.00		

PDL_history	Score 0	9	1	25.00	8	47.10	4.118	0.128
	Score 1	5	0	0.00	5	29.40		
	Score 2	0	0	0.00	0	0.00		
	Score 3	7	3	75.00	4	23.50		

Plaque_status	Score 0	4	0	0.00	4	23.50	5.507	0.138
	Score 1	9	1	25.00	8	47.10		
	Score 2	6	3	75.00	3	17.60		
	Score 3	2	0	0.00	2	11.80		

Gingival_status	Score 0	11	0	0.00	11	64.70	5.868	0.053
	Score 1	4	2	50.00	2	11.80		
	Score 2	6	2	50.00	4	23.50		
	Score 3	0	0	0.00	0	0.00		

Diabetes	Absent	21	4	100.00	17	100.00		
	Present	0	0	0.00	0	0.00		

Smoking	Absent	17	2	50.00	15	88.20	3.07	0.08
	Present	4	2	50.00	2	11.80		

Residual_cement	Absent	21	4	100.00	17	100.00		
	Present	0	0	0.00	0	0.00		

Occlusal_overload	Absent	19	3	75.00	16	94.10	1.373	0.241
	Present	2	1	25.00	1	5.90		

Width_keratinized_gingiva	Absent	16	2	50.00	14	82.40	1.868	0.172
	Present	5	2	50.00	3	17.60		

Others	Absent	15	1	25.00	14	82.40	5.219	**0.022**
	Present	6	3	75.00	3	17.60		

Result	Low	15	1	25.00	14	82.40	5.219	**0.022**
	Moderate	6	3	75.00	3	17.60		

## Data Availability

The data used to support the findings of this study are included within the article.
